# Enhancing the Electromechanical Coupling in Soft Energy Harvesters by Using Graded Dielectric Elastomers

**DOI:** 10.3390/mi12101187

**Published:** 2021-09-30

**Authors:** Lingling Chen, Shengyou Yang

**Affiliations:** 1 Department of Engineering Mechanics, Shandong University, Jinan 250061, China; 202120659@mail.sdu.edu.cn; 2 Suzhou Research Institute, Shandong University, Suzhou 215123, China

**Keywords:** dielectric elastomer, electromechanical coupling, graded film, energy harvesting, specific energy

## Abstract

Soft dielectric elastomers can quickly achieve large deformations when they are subjected to electromechanical loads. They are widely used to fabricate a number of soft functional devices. However, the functions of soft devices are limited to the failure modes of soft dielectric elastomers. In this paper, we use graded dielectric elastomers to produce a soft energy harvester with a strong ability of energy harvesting. Compared to the conventional energy harvester with homogeneous dielectric films, our new energy harvester is made of graded elastomers and can increase both the specific energy from 2.70 J/g to 2.93 J/g and the maximum energy from 6.3 J/g to 8.6 J/g by just using a stiffer outer radius. By optimizing the material parameters in graded dielectric films, the soft energy harvester can reach better performance, and our results can provide guidance for designing powerful energy harvesters.

## 1. Introduction

Rapid technological advance has greatly facilitated people’s lives while consuming massive resources. To ensure the sustainable development of human life, researchers have been continuously exploring and researching new energy storage materials. For a long period of time, the vast majority of research looks toward new energy storage materials in the field of hard materials for answers. Among them, piezoelectric ceramic (PZT) is one of the most widely used materials in the field of rigid materials for energy harvesters [1,2,3,4,5,6,7]. With the rapid development of network and communication technologies, there comes a higher requirement on the energy storage materials and devices, for example, flexoelectricity-based energy harvesting is an alternative to piezoelectrics in nanoscale [8,9]. In other words, traditional hard materials cannot meet the needs of social production and daily life. A lot of researchers turned to soft materials for answers.

Soft materials such as soft dielectric elastomers have many dynamic features such as high resilience, low moduli and viscoelasticity, which are superior to other hard materials. Thus, it shows wide application prospects in the fields of military, aerospace, communications, instrumentation, machinery and medical equipment sectors, etc. [10,11,12,13,14,15,16,17,18]. However, soft dielectrics under applied the mechanical and electric loads can achieve large deformation, which invariably results in various types of electromechanical failures. Failures further results in low convertible maximum energy, and then the result is that the application of functional devices of soft dielectrics is limited. Some standard modes of failure [19,20,21,22,23,24,25,26,27,28,29,30] include electromechanical instability, rupture by stretch and electric breakdown, among others.

Our previous study [31] has shown large deformation, pull-in instability and electro-actuation of a graded circular dielectric plate subjected to the in-plane mechanical load and the applied electric load in the thickness direction. These results verify that the ability to sustain a high electric field or a large deformation in a stiff or soft homogeneous circular dielectric plate can be achieved by only using a graded circular dielectric plate. We only have to partly change the modulus of a circular film with a stiff or soft outer region. In this paper, we aim to use the graded materials to enhance the functionality of a soft energy harvester. In detail, we would like to increase the specific energy, the output voltage and the maximum energy of a soft energy harvester by using graded dielectric films.

The outline of this work is as follows. In Section 2, we illustrate a typical energy harvester, including the electromechanical behaviors and steps of energy harvesting. In Section 3, we revisit the electromechanical couplings in a circular film of homogeneous dielectric elastomers. The equilibrium states and four modes of failure are shown mathematically. We further illustrate the electromechanical behaviors on both the stress vs. stretch plane and the nominal electric field vs. nominal electric displacement plane. In contrast, Section 4 focuses on the electromechanical behaviors of a circular film of graded dielectric elastomers. We normalize the equilibrium equations and equations of four modes of failure. We then show graphically the electromechanical couplings in a graded film. Results and discussions are given in Section 5. Conclusions are given in Section 6.

## 2. A Typical Energy Harvester of Dielectric Elastomers

**Figure 1:**
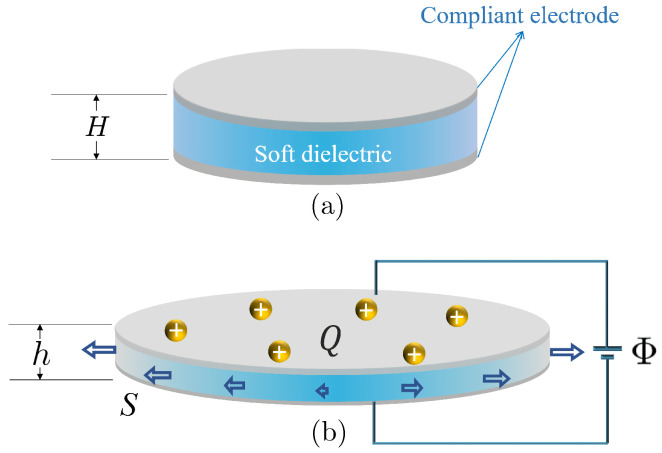
Schematic of the deformation of a circular film of soft dielectrics subjected to an electric voltage Φ and a surrounding dead load *S*. The circular film is coated with two compliant electrodes on the upper and bottom surfaces. (**a**) Undeformed circular film with radius *B* and thickness *H*. (**b**) Deformed circular film with thickness *h*. Each electrode gains an electric charge of magnitude *Q*.

Consider a simple but commonly used device shown in Figure 1. The soft device consists of a circular dielectric film coated with compliant electrodes on its upper and bottom surfaces. By applying a voltage Φ in the thickness direction and a radial dead load *S* on the lateral surface, the soft film will expand its in-plane area and decrease its thickness due to the highly nonlinear electromechanical coupling, i.e., the film thickness will decrease from *H* to *h* and then its in-plane area increases because of the constraint of incompressibility. In addition, each compliant electrode gains an amount of electric charges *Q* during the electromechanical loading process. Therefore, we can simply consider the device shown in Figure 1 as a soft capacitor that can store a certain amount of mechanical and electric energy.

In general, the capacity of a parallel plate capacitor, made of hard dielectric materials, mainly depends on three key factors including the separation between the two plates, the area of the plates, and the permittivity of dielectric materials enclosed by the two plates. For a hard dielectric capacitor, we usually ignore its deformation during the stored procedure for electric energy, and then its capacity can be seen as a constant. However, the capacity in a soft capacitor is very different from the capacity in a hard capacitor. For a soft dielectric capacitor, its thickness decreases, and the in-plane area increases; therefore, the capacity of soft dielectric capacitor increases due to its expansion in area and its reduction in thickness.

Due to electromechanical coupling, the soft device shown in Figure 1 can be seen as either a soft actuator with large actuation or a charge carrier with the property of varied capacity. Moreover, the unique properties render the soft device as a good candidate for the design of a charge pumper that can pump electric charges from low voltage to high voltage.

**Figure 2:**
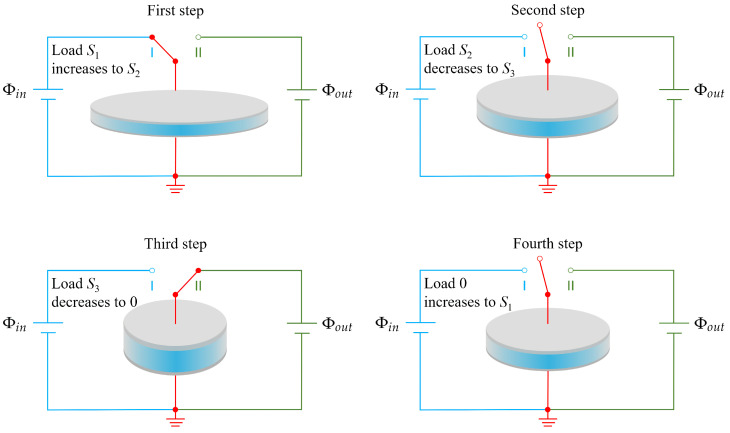
Schematic of the performance of a soft energy harvester that is able to pump electric charges from low voltage ($\Phi_{in}$) to high voltage ($\Phi_{out}$) by using a switch.

Let us look at the electric system shown in Figure 2. The soft dielectric film can be connected to either a power source with low voltage ($\Phi_{in}$) or a power source with high voltage ($\Phi_{out}$) by using a switch. We will show how to pump electric charges from low voltage to high voltage. In the first step, the switch is switched on to I; meanwhile, we gradually increase the applied load *S*. During this process, the applied voltage on the film is constant ($\Phi_{in}$), and the area of film increases; therefore, the dielectric film can carry more charges. In the second step, after the film gains a certain amount of charges ($Q_{in}$), we disconnect the switch, and the voltage on the film is equal to $\Phi_{in}$ at this moment. We then gradually decrease the applied load and the area of the film decreases, which makes the capacity of the soft capacitor decrease. Since the charges on the surface are isolated and there is a decrease in capacity, the voltage difference between the upper and bottom surfaces increases. In the third step, after the voltage increases to a threshold that is larger than the output voltage $\Phi_{out}$, we connect the switch to II, and then the charges proceed to power source II. In the fourth step, we disconnect the switch. The four stages form a cycle. With the input mechanical energy and the output electric energy, the soft device can be used as an energy harvester.

The aforementioned four steps involve highly nonlinear electromechanical couplings including large deformation, pull-in instability, lose-of-tension, rupture by stretch and electric breakdown. These nonlinear behaviors and failure modes directly affect the functionality of the soft energy harvester, including the specific energy during a cycle, the output voltage and the maximum energy that can be harvested. In this paper, we would like to use graded dielectric films to enhance the functionality and the efficiency of a soft energy harvester.

## 3. Electromechanical Couplings in a Circular Film of Homogeneous Dielectric Elastomers

Consider the circular film of dielectric elastomers shown in Figure 1. The nominal electric field $\tilde{E}$ in the thickness direction is defined as $\tilde{E}=\Phi /H$, and the nominal electric displacement $\tilde{D}$ is $\tilde{D}=Q/\left(\pi B^{2}\right)$, where *H* is the thickness and *B* is the radius before the deformation. In this section, we consider a homogeneous dielectric elastomer for which its modulus and material permittivity are constant. If we take the neo-Hookean model of soft materials, for example, the shear modulus μ and material permittivity ε of the film are independent of the coordinates.

For a given pair of electromechanical loads $\left(\tilde{E},S\right)$, the film thickness decreases from *H* to *h* homogeneously. We remark that α is the stretch in the thickness direction of the dielectric film, and the following is the case

(1) $$\alpha =\frac{h}{H},$$

where *h* is the film thickness after the deformation. If we define the two principal in-plane stretches as λ, we have the relation $\alpha =\lambda^{-2}$ for incompressible elastomers due to the constraint of incompressibility, i.e., $\lambda^{2}\alpha =1$.

### 3.1. Equilibrium States

When the circular film is at equilibrium, we have the two governing equations [21,31,32]:

(2) $$\frac{\tilde{E}}{\sqrt{\mu /\varepsilon }}=\sqrt{\left(\alpha -\alpha^{4}\right)-\frac{S}{\mu }\alpha^{3/2}}$$

and

(3) $$\frac{\tilde{E}}{\sqrt{\mu /\varepsilon }}=\alpha^{2}\frac{\tilde{D}}{\sqrt{\varepsilon \mu }}.$$

Note that (2) is the relation between the nominal electric field $\tilde{E}$, the in-plane dead load *S* and the stretch α in the thickness direction. If we replace α by $\lambda^{-2}$ in (2), we exactly obtain Equation ${\left(8\right)}_{2}$, which is $\frac{\tilde{E}}{\sqrt{\mu /\varepsilon }}=\sqrt{\lambda^{-2}-\lambda^{-8}-\frac{s}{\mu }\lambda^{-3}}$, in the work by Zhao and Suo [21].

### 3.2. Four Modes of Failure

When the circular film is subjected to electromechanical loads, there may exist multiple failure modes that impede the functionality of soft energy harvester. We now discuss the main four failure modes and their conditions.

1. **Electromechanical instability (EMI)**: When the electromechanical loads reach the threshold, the condition for the onset of electromechanical instability is as follows [31]:
  (4) $$\frac{3}{2}\frac{S}{\mu }\alpha^{1/2}-\left(1-4\alpha^{3}\right)=0.$$
  The stretch α also has to satisfy the equilibrium Equations (2) and (3).
2. **Loss of tension (LT)**: When the nominal stress *S* in (2) becomes zero, it is the so-called state of loss of tension, and the following is the case
  (5) S=0.
3. **Rupture by stretch (RS)**: The film ruptures when the in-plane stretch λ, i.e., $\lambda =\alpha^{-1/2}$, reaches a critical value, and the following is the case
  (6) $$\lambda =\lambda_{R}.$$
  Usually, the critical stretch $\lambda_{R}$ for rupture in the experiment [19] of equal biaxial stretch is suggested as $\lambda_{R}\leq 6$. In this paper, we chose the same value $\lambda_{R}=5$ as that used in the work [32] in order to show how the graded modulus affects energy conversion. Note that the stretch $\lambda =\alpha^{-1/2}$ here is governed by the equilibrium Equations (2) and (3).
4. **Electric breakdown (EB)**: When the true electric field E=Φ/h, i.e., $E=\Phi /h=\alpha^{-1}\Phi /H=\alpha^{-1}\tilde{E}$, reaches a critical value ($E_{\mathrm{EB}}$), the dielectric film accompanies the occurrence of electric breakdown, and the following is the case
  (7) $$\alpha^{-1}\tilde{E}=E_{\mathrm{EB}}.$$
  Based on the existing experiments [10], the critical electric field for the onset of EB is chosen as $E_{\mathrm{EB}}=3\times 10^{8}\;V/m$. Other material parameters used in the numerical calculations are $\mu =10^{6}\;N\cdot m^{-2}$ and $\varepsilon =3.54\times 10^{-11}\;F\cdot m^{-1}$, as well as the mass density $\rho =1000\;\mathrm{kg}\cdot m^{-3}$.

### 3.3. In-Plane Stress vs. Stretch Curves

On the stress vs. stretch plane (the $\frac{S}{\mu }$ vs. $\alpha^{-1/2}$ plane), we have to plot five curves that correspond to the purely mechanical loading process and other four failure modes. The area enclosed by the five curves can be regarded as a safe area within which the soft energy harvester works well without any damage. We would like to show how much energy can be enclosed by the five curves on the stress vs. stretch plane.

1. **Purely mechanical (PM) curve**: By setting $\tilde{E}=0$ in (2), we have the following PM curve
  (8) $$\frac{S}{\mu }=\alpha^{-1/2}-\alpha^{5/2}.$$
2. **EMI curve**: By the stability condition (4), we have the EMI curve
  (9) $$\frac{S}{\mu }=\frac{2}{3}\left(\alpha^{-1/2}-4\alpha^{5/2}\right).$$
3. **LT curve**: The LT curve at S=0 actually corresponds to the horizontal axis.
4. **RS curve**: By (6), the RS curve corresponds to the vertical line $\alpha^{-1/2}=\lambda_{R}=5$.
5. **EB curve**: By (7), we have $\tilde{E}=\alpha E_{\mathrm{EB}}$. Replacing $\tilde{E}$ in (2) by $\alpha E_{\mathrm{EB}}$, we then have the EB curve
  (10) $$\frac{S}{\mu }=\alpha^{-1/2}-\alpha^{5/2}-\alpha^{1/2}{\left(\frac{E_{\mathrm{EB}}}{\sqrt{\mu /\varepsilon }}\right)}^{2}.$$

### 3.4. Nominal Electric Field vs. Nominal Electric Displacement Curves

Corresponding to the five curves on the stress vs. stretch plane, we have to plot five corresponding curves on the nominal electric field vs. nominal electric displacement plane (the $\frac{\tilde{E}}{\sqrt{\mu /\varepsilon }}$ vs. $\frac{\tilde{D}}{\sqrt{\varepsilon \mu }}$ plane). The relation between $\frac{\tilde{E}}{\sqrt{\mu /\varepsilon }}$ and $\frac{\tilde{D}}{\sqrt{\varepsilon \mu }}$ in (3) directly depends on the stretch α. Therefore, we have to determine the expression of α in each curve.

(1) **Purely mechanical (PM) curve**: By setting $\tilde{E}=0$ in (3), the PM curve corresponds to the *origin* of the $\frac{\tilde{E}}{\sqrt{\mu /\varepsilon }}$ vs. $\frac{\tilde{D}}{\sqrt{\varepsilon \mu }}$ plane, i.e., $\left(\frac{\tilde{E}}{\sqrt{\mu /\varepsilon }},\frac{\tilde{D}}{\sqrt{\varepsilon \mu }}\right)=\left(0,0\right)$.
(2) **EMI curve**: By (3), we have $\alpha ={\left(\frac{\tilde{E}}{\sqrt{\mu /\varepsilon }}/\frac{\tilde{D}}{\sqrt{\varepsilon \mu }}\right)}^{1/2}$. By substituting the expression of α into (2) and (4) and then rearranging the results, we obtain the EMI curve
  (11) $$\frac{\tilde{E}}{\sqrt{\mu /\varepsilon }}={\left[3{\left(\frac{\tilde{D}}{\sqrt{\varepsilon \mu }}\right)}^{1/2}-5{\left(\frac{\tilde{D}}{\sqrt{\varepsilon \mu }}\right)}^{-3/2}\right]}^{-2/3}.$$
  Actually, (11) is the same as Equation (7) in the work by Koh et al. [32].
(3) **LT curve**: By setting S=0 in (2), we have the relation $\frac{\tilde{E}}{\sqrt{\mu /\varepsilon }}=\sqrt{\alpha -\alpha^{4}}$. By substituting the expression of α from (3) into the relation, we have the LT curve
  (12) $$\frac{\tilde{E}}{\sqrt{\mu /\varepsilon }}={\left({\left(\frac{\tilde{D}}{\sqrt{\varepsilon \mu }}\right)}^{1/2}+{\left(\frac{\tilde{D}}{\sqrt{\varepsilon \mu }}\right)}^{-3/2}\right)}^{-2/3}.$$
  Moreover, (12) is the same as the Equation (8) in the work by Koh et al. [32].
(4) **RS curve**: The RS curve corresponds to the vertical line $\alpha^{-1/2}=\lambda_{R}=5$ in (6). Together with the expression of α from (3), we obtain the RS curve
  (13) $$\frac{\tilde{E}}{\sqrt{\mu /\varepsilon }}=\lambda_{R}^{-4}\cdot \frac{\tilde{D}}{\sqrt{\varepsilon \mu }},$$
  which is the same as Equation (9) in the work by Koh et al. [32].
(5) **EB curve**: By (7), we have $\alpha =\tilde{E}E_{\mathrm{EB}}^{-1}$. Replacing α in (3) by $\tilde{E}E_{\mathrm{EB}}^{-1}$, we have the EB curve
  (14) $$\frac{\tilde{E}}{\sqrt{\mu /\varepsilon }}=\frac{E_{\mathrm{EB}}^{2}}{\mu /\varepsilon }\cdot {\left(\frac{\tilde{D}}{\sqrt{\varepsilon \mu }}\right)}^{-1},$$
  which is the same as Equation (5) in the work by Koh et al. [32].

### 3.5. Energy of Conversion

**Figure 3:**
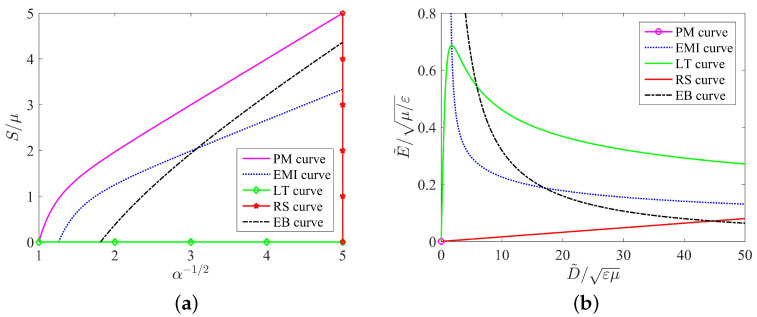
A thermodynamic state of the dielectric film is represented by (**a**) a point in the stress-stretch plane or (**b**) a point in the nominal electric field-nominal electric displacement plane.

This paper attempts to solve the dielectric elastomer transducer problem of nonlinear systems by using a point in the stress–stretch plane or a point in the nominal electric field-nominal electric displacement plane, for example, with two degrees of freedom. By the two equilibrium equations of (2) and (3), one may not only clarify the independent variable regarding the dependent variable influence size and the tendency but also may seek its best energy capture condition. Each mechanism of failure as one curve can be represented in the stress–stretch plane (see Figure 3a) or the nominal electric field-nominal electric displacement plane (see Figure 3b).

In Figure 3a, we take the radial stretch $\alpha^{-1/2}$ as the x-axis and the dead load S/μ as the y-axis. We plot five curves including the purely mechanical (PM) curve governed by (8), the electromechanical instability (EMI) curve governed by (9), the loss of tension (LT) curve, the rupture by stretch (RS) curve, and the electric breakdown (EB) curve governed by (10). In contrast, the corresponding five curves on the nominal electric field-nominal electric displacement plane are shown in Figure 3b. The purely mechanical (PM) curve now becomes the origin, the electromechanical instability (EMI) curve is governed by (11), the loss of tension (LT) curve is governed by (12), the rupture by stretch (RS) curve is governed by (13), and the electric breakdown (EB) curve is governed by (14).

In either Figure 3a or Figure 3b, the area enclosed by the five curves is important for the energy harvester. The area directly determines how much energy can be harvested by using the soft energy harvester during a loading-unloading process. Importantly, each point in the enclosed area corresponds to a safe state of the circular dielectric film, i.e., the film has no damage. However, for any point of the enclosed area, the dielectric film may encounter at least one failure mode that would damage the film, which makes the soft energy harvester fail. Therefore, we have to find an effective method to increase the area enclosed by the curves if we would like to improve the functionality of a soft energy harvester.

## 4. Electromechanical Couplings in a Circular Film of Graded Dielectric Elastomers

In order to enhance the performance of a soft energy harvester, we used graded dielectric materials rather than homogeneous dielectric materials to fabricate the circular film shown in Figure 1. The graded material definitely changes the electromechanical coupling behaviors shown in Figure 3; therefore, the ability of energy harvester can be either enhanced or weakened. More exactly, the five curves in either Figure 3a or Figure 3b would be changed by using a graded dielectric elastomer, and the corresponding area enclosed by the five curves changes. We would like to find an effective method to increase the enclosed area.

Consider a circular film with varied modulus in the radial direction, i.e., the shear modulus μ(R) is no longer a constant but varies along with the radius *R*. As stated in a recent paper by Chen et al. [31], the electromechanical behaviors directly depend on the shear modulus μ(B) of a circular film at its outer radius R=B. For example, the equilibrium Equation (36) and the instability condition (38) in [31] only depend on the shear modulus at the outer radius.

In this paper, we only consider varied shear modulus μ(R) in the graded dielectric film. All other material parameters of the graded dielectric elastomer, including the dielectric permittivity ε, the rupture of stretch $\lambda_{R}$ and the electric breakdown $E_{\mathrm{EB}}$, are independent of the coordinates and are the same as that of a homogeneous film. For a graded dielectric film with shear modulus $\mu_{\gamma }=\mu \left(B\right)$ at its outer radius, we briefly list the equilibrium equations and the governing equations of the four modes of failure in the following sections.

### 4.1. Equilibrium Equation and Four Modes of Failure

Consider a graded dielectric film with shear modulus $\mu_{\gamma }$ at its outer radius R=B. Similar to the equilibrium Equations (2) and (3) for a homogeneous material with shear modulus μ, the equilibrium equations of the graded film are as follows:

(15) $$\frac{\tilde{E}}{\sqrt{\mu_{\gamma }/\varepsilon }}=\sqrt{\left(\alpha -\alpha^{4}\right)-\frac{S}{\mu_{\gamma }}\alpha^{3/2}}\;\mathrm{and}\;\frac{\tilde{E}}{\sqrt{\mu_{\gamma }/\varepsilon }}=\alpha^{2}\frac{\tilde{D}}{\sqrt{\varepsilon \mu_{\gamma }}},$$

The four modes of failure are determined by the following

(16) $$\left\{\begin{array}{c} \mathrm{EMI}:\;\frac{3}{2}\frac{S}{\mu_{\gamma }}\alpha^{1/2}-\left(1-4\alpha^{3}\right)=0,\\ \mathrm{LT}:\;S=0,\\ \mathrm{RS}:\;\;\;\alpha^{-1/2}=\lambda_{R},\\ \mathrm{EB}:\;\;\;\alpha^{-1}\tilde{E}=E_{\mathrm{EB}}. \end{array}\right.$$

### 4.2. Curves on the Two Planes

Similar to the five curves in Section 3.3, the five curves of graded dielectric film on the stress $\frac{S}{\mu_{\gamma }}$ vs. stretch $\alpha^{-1/2}$ plane are listed as follows:

(17) $$\left\{\begin{array}{c} \mathrm{PM}:\frac{S}{\mu_{\gamma }}=\alpha^{-1/2}-\alpha^{5/2},\\ \mathrm{EMI}:\frac{S}{\mu_{\gamma }}=\frac{2}{3}\left(\alpha^{-1/2}-4\alpha^{5/2}\right),\\ \mathrm{LT}:\mathrm{the}\;\mathrm{horizontal}\;\mathrm{axis},\\ \mathrm{RS}:\alpha^{-1/2}=\lambda_{R}=5,\\ \mathrm{EB}:\frac{S}{\mu_{\gamma }}=\alpha^{-1/2}-\alpha^{5/2}-\alpha^{1/2}{\left(\frac{E_{\mathrm{EB}}}{\sqrt{\mu_{\gamma }/\varepsilon }}\right)}^{2}. \end{array}\right.$$

On the $\frac{\tilde{E}}{\sqrt{\mu_{\gamma }/\varepsilon }}$ vs. $\frac{\tilde{D}}{\sqrt{\varepsilon \mu_{\gamma }}}$ plane, the five curves of graded dielectric film are summarized as follows:

(18) $$\left\{\begin{array}{c} \mathrm{PM}:\mathrm{the}\;\mathrm{origin}\;\left(0,0\right),\\ \mathrm{EMI}:\frac{\tilde{E}}{\sqrt{\mu_{\gamma }/\varepsilon }}={\left[3{\left(\frac{\tilde{D}}{\sqrt{\varepsilon \mu_{\gamma }}}\right)}^{1/2}-5{\left(\frac{\tilde{D}}{\sqrt{\varepsilon \mu_{\gamma }}}\right)}^{-3/2}\right]}^{-2/3},\\ \mathrm{LT}:\;\frac{\tilde{E}}{\sqrt{\mu_{\gamma }/\varepsilon }}={\left({\left(\frac{\tilde{D}}{\sqrt{\varepsilon \mu_{\gamma }}}\right)}^{1/2}+{\left(\frac{\tilde{D}}{\sqrt{\varepsilon \mu_{\gamma }}}\right)}^{-3/2}\right)}^{-2/3},\\ \mathrm{RS}:\;\frac{\tilde{E}}{\sqrt{\mu_{\gamma }/\varepsilon }}=\lambda_{R}^{-4}\cdot \frac{\tilde{D}}{\sqrt{\varepsilon \mu_{\gamma }}},\\ \mathrm{EB}:\;\frac{\tilde{E}}{\sqrt{\mu_{\gamma }/\varepsilon }}=\frac{E_{\mathrm{EB}}^{2}}{\mu_{\gamma }/\varepsilon }\cdot {\left(\frac{\tilde{D}}{\sqrt{\varepsilon \mu_{\gamma }}}\right)}^{-1}. \end{array}\right.$$

### 4.3. Scaling

We adopt a scaling in which (critical) nominal electric fields and electric breakdowns are measured relative to $\sqrt{\mu /\varepsilon }$, nominal electric displacement is measured relative to $\sqrt{\varepsilon \mu }$ and force density and shear modulus are measured relative to μ. This results in the following dimensionless measures

(19) $$\bar{E}=\frac{\tilde{E}}{\sqrt{\mu /\varepsilon }},\;\frac{E_{\mathrm{EB}}}{\sqrt{\mu /\varepsilon }}=\eta ,\;\bar{D}=\frac{\tilde{D}}{\sqrt{\varepsilon \mu }},\;\bar{S}=\frac{S}{\mu },\;\gamma =\frac{\mu_{\gamma }}{\mu }.$$

We remark that the ratio γ is the modulus $\mu_{\gamma }$ of the graded film at its outer radius to the modulus μ of the homogeneous film. In the case of γ>1, the graded film has a stiffer modulus compared to the homogeneous film and vice versa.

By using the dimensionless parameters in (19), we further have the following

(20) $$\frac{\tilde{E}}{\sqrt{\mu_{\gamma }/\varepsilon }}=\frac{\bar{E}}{\sqrt{\gamma }},\;\frac{E_{\mathrm{EB}}}{\sqrt{\mu_{\gamma }/\varepsilon }}=\frac{\eta }{\sqrt{\gamma }},\;\frac{\tilde{D}}{\sqrt{\varepsilon \mu_{\gamma }}}=\frac{\bar{D}}{\sqrt{\gamma }},\;\frac{S}{\mu_{\gamma }}=\frac{\bar{S}}{\gamma }.$$

#### 4.3.1. Dimensionless Equations of Equilibrium and Four Modes of Failure

By using (20), the equilibrium Equation (15) of the graded film can be written as the following

(21) $$\bar{E}=\sqrt{\gamma \left(\alpha -\alpha^{4}\right)-\bar{S}\alpha^{3/2}}\;\mathrm{and}\;\bar{E}=\alpha^{2}\bar{D},$$

Equation (16) of four modes of failure can be rearranged as follows:

(22) $$\left\{\begin{array}{c} \mathrm{EMI}:\;\frac{3}{2}\bar{S}\alpha^{1/2}-\gamma \left(1-4\alpha^{3}\right)=0,\\ \mathrm{LT}:\;\bar{S}=0,\\ \mathrm{RS}:\;\;\;\alpha^{-1/2}=\lambda_{R},\\ \mathrm{EB}:\;\;\;\alpha^{-1}\bar{E}=\eta . \end{array}\right.$$

#### 4.3.2. Dimensionless Equations of Five Curves

Similarly, the five curves (17) on the $\bar{S}$ vs. $\lambda =\alpha^{-1/2}$ plane are as follows:

(23) $$\left\{\begin{array}{c} \mathrm{PM}:\bar{S}=\gamma \left(\lambda -\lambda^{-5}\right),\\ \mathrm{EMI}:\bar{S}=\frac{2}{3}\gamma \left(\lambda -4\lambda^{-5}\right),\\ \mathrm{LT}:\mathrm{the}\;\mathrm{horizontal}\;\mathrm{axis},\\ \mathrm{RS}:\lambda =\lambda_{R}=5,\\ \mathrm{EB}:\bar{S}=\gamma \left(\lambda -\lambda^{-5}\right)-\lambda^{-1}\eta^{2}, \end{array}\right.$$

The five curves (18) on $\bar{E}$ vs. $\bar{D}$ plane are as follows:

(24) $$\left\{\begin{array}{c} \mathrm{PM}:\mathrm{the}\;\mathrm{origin}\;\left(0,0\right),\\ \mathrm{EMI}:\bar{E}=\bar{D}{\left(3{\bar{D}}^{2}\gamma^{-1}-5\right)}^{-2/3},\\ \mathrm{LT}:\;\bar{E}=\bar{D}{\left({\bar{D}}^{2}\gamma^{-1}+1\right)}^{-2/3},\\ \mathrm{RS}:\;\bar{E}=\lambda_{R}^{-4}\cdot \bar{D},\\ \mathrm{EB}:\;\bar{E}=\eta^{2}\cdot {\bar{D}}^{-1}. \end{array}\right.$$

### 4.4. Energy of Conversion

If we consider the case of γ=1 in Section 4.3.1 and Section 4.3.2, then the electromechanical responses reduce to those of the homogeneous film with shear modulus μ. We show the curves with three typical values of γ=0.5,1,2; therefore, the effects of graded materials on the electromechanical behaviors can be visualized apparently.

**Figure 4:**
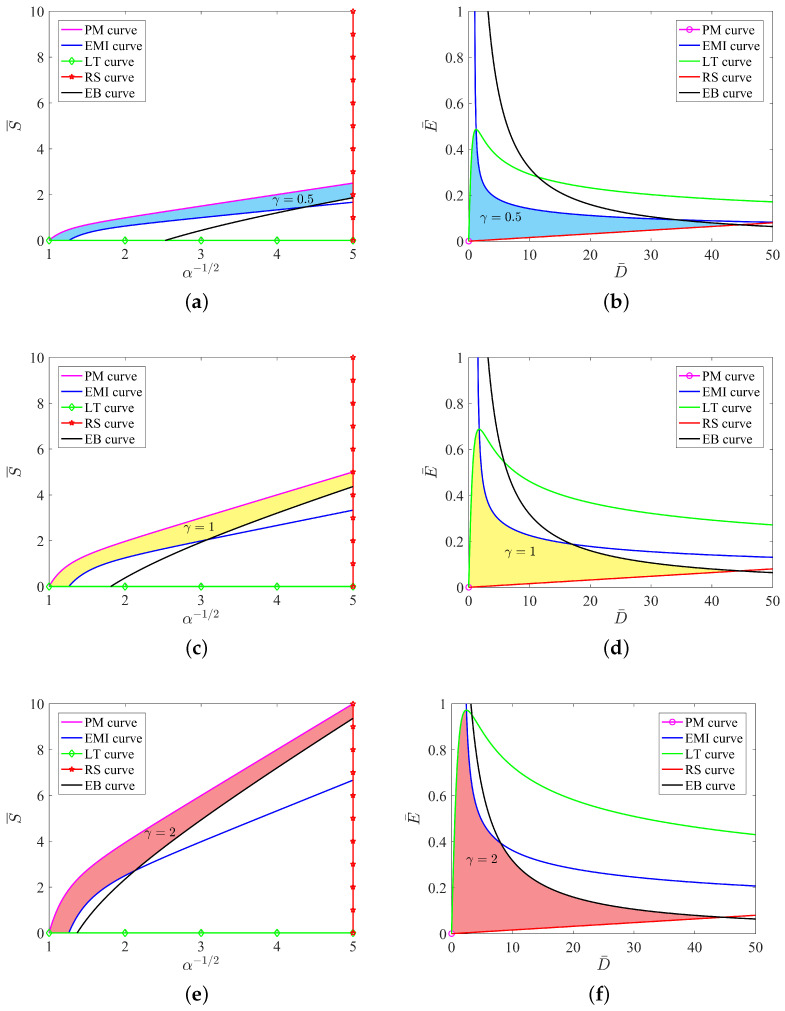
Five curves on the stress $\bar{S}$ vs. stretch $\lambda =\alpha^{-1/2}$ plane and the dimensionless electric field $\bar{E}$ vs. $\bar{D}$ plane under different ratios γ=0.5,1,2. The shaded area in each figure is the maximum specific energy that can be harvested. γ=0.5 in (**a**,**b**), γ=1 in (**c**,**d**), γ=2 in (**e**,**f**).

We first consider the effects of γ on the five curves on the stress $\bar{S}$ vs. stretch $\lambda =\alpha^{-1/2}$ plane. The governing equations of the five curves are expressed in (23). In Figure 4a,c,e, it is clear that the increase in the ratio γ has no effects on the two failure modes, loss of tension and rupture by stretch. This is because the LT curve corresponds to the horizontal axis and the RS curve is a vertical line, see (23). However, the varied ratio γ in (23) inevitably changes the purely mechanical (PM) curve, the electromechanical instability (EMI) curve and the electric breakdown (EB) curve. As shown in Figure 4a,c,e, we take the PM curve $\bar{S}=\gamma \left(\lambda -\lambda^{-5}\right)$ as an example. At a fixed value of the stretch λ, the increase in γ linearly increases the value of the mechanical load $\bar{S}$. Such a linear behavior can also be found in the EMI curve $\bar{S}=\frac{2}{3}\gamma \left(\lambda -4\lambda^{-5}\right)$ by increasing the ratio γ. Unfortunately, the linear behavior described in either the PM curve or the EMI curve is not true in the EB curve. The reason is that the EB curve $\bar{S}=\gamma \left(\lambda -\lambda^{-5}\right)-\lambda^{-1}\eta^{2}$ in (23) is no longer a linear function of γ.

In contrast, we also show the effects of γ on the five curves in the $\bar{E}$ vs. $\bar{D}$ plane in Figure 4b,d,f. The governing equations of the five curves are given by (24). The change of the ratio γ has no effects on the PM curve (the origin), the RS curve and the EB curve. In other words, those three curves are independent of the value of γ on the $\bar{E}$ vs. $\bar{D}$ plane. In contrast, the EMI curve and the LT curve depend on the value of γ non-linearly.

In Figure 4, we also note that the area enclosed by the five curve in the $\bar{E}$ vs. $\bar{D}$ plane changes with the increase in the ratio γ. It is known that the shaded area in the allowable state surrounded by various failure modes can be expressed as the *maximal specific energy*. It can be observed in Figure 4 that as the ratio γ increases, the shaded area increases, indicating that the maximum conversion energy increases. As mentioned in the work [32], four modes of failure result in a maximal specific energy of 6.3 J/g, corresponding to γ=1 in Figure 4. Interestingly, the modes of failure admit a maximal specific energy that can be as high as 8.6 J/g at γ=2. Based on the discussion of Figure 4, we can increase the shadow area in the allowable state by changing the shear modulus of the material, thereby obtaining greater energy conversion efficiency.

## 5. Results and Discussion

### 5.1. Specific Energy and Output Voltage

**Figure 5:**
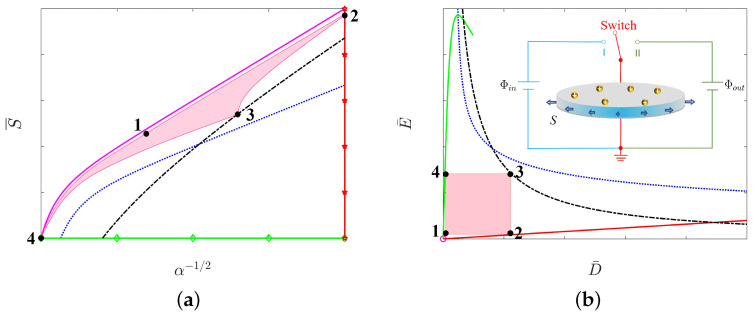
The cycle of energy harvesting is represented by the boundary of a shaded area in either (**a**) the dimensionless stress vs. strain plane or (**b**) the nominal electric field vs. nominal electric displacement plane. In (**b**), four sides 12, 23, 34 and 41 of the rectangle represent different steps of the process of energy harvesting. The detailed four steps of the soft energy harvester have been illustrated in Figure 2.

In the area enclosed by the five curves on the dimensionless electric field $\bar{E}$ vs. $\bar{D}$ plane, we plot a rectangle for which its four vertices are represented, respectively, by 1, 2, 3 and 4 in Figure 5b. The corresponding vertices on the dimensionless stress $\bar{S}$ vs. the stretch plane are shown in Figure 5a. Note that the vertices 2, 3 and 4 are on the curves. Each side of the rectangle in Figure 5b represents a step of the process of energy harvesting. In particular, the four sides of the rectangle form a cycle of the energy harvesting by using the soft energy harvester. We also plot the soft energy harvester in the neighborhood of the rectangle to show how the energy harvester works in each step.

In Figure 5b, the nominal electric field $\bar{E}$ on the side 12 is constant, which can also be presented by an input voltage $\Phi_{in}$. In this process, from vertex 1 to vertex 2, the switch is on I, and the nominal electric field $\bar{E}$ (the input voltage $\Phi_{in}$) is constant, but the applied load increases in order to make the soft film expand and gain a certain amount of charges. In the second step, from vertex 2 to vertex 3, we disconnect the switch, and the nominal electric displacement $\bar{D}$ (the gained charges *Q*) is constant, but the applied load decreases in order to make the soft film shrink and obtain a high output voltage $\Phi_{out}$. In the third step, from vertex 3 to vertex 4, the switch is on II, and the nominal electric field $\bar{E}$ (the output voltage $\Phi_{out}$) is constant, but the applied load decreases in order to make the soft film shrink and export a number of charges. In the fourth step, from vertex 3 to vertex 4, we disconnect the switch, and the nominal electric displacement $\bar{D}$ is constant, but the applied load decreases in order to make the soft film return to the vertex 1. Thus, the cycle is complete, and a certain number of charges have been pumped from low voltage $\Phi_{in}$ to high voltage $\Phi_{out}$.

**Figure 6:**
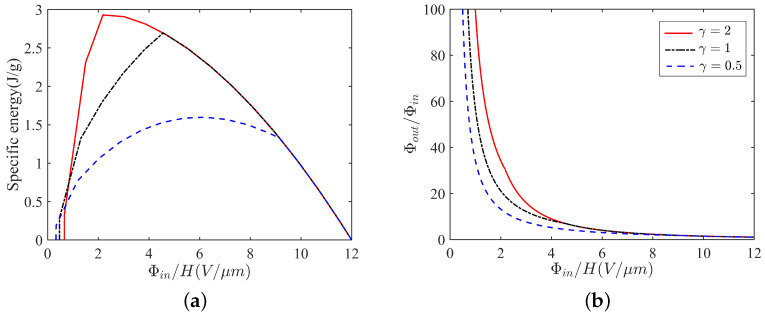
Specific energy (**a**) and amplification of voltage (**b**) for cycles represented by various rectangles in Figure 5 by using different ratios γ=0.5,1,2.

We remark that there are two values that should be highlighted in Figure 5. One is the area of the rectangle, which is called the *specific energy* that can be harvested during a cycle. The other is the output voltage $\Phi_{out}$, which corresponds to the nominal electric field on the side 34 that is the amplification of voltage. For example, if we apply an input voltage $\Phi_{in}$ on the energy harvester, we would like to know how much the specific energy can be obtained and to what extent the output voltage can be amplified during a cycle by using a soft energy harvester. Can a graded film increase the two values of a soft energy harvester? We provide the answers in Figure 6.

Figure 6a shows the specific energy (the area of the rectangle in Figure 5) generated per cycle of operation versus the input nominal electric field ($\bar{E}$ on side 12 in Figure 5). Material parameters [10,25,32] used in the plots are chosen as $E_{\mathrm{EB}}=3\times 10^{8}\;V/m$, $\mu =10^{6}\;N\cdot m^{-2}$ and $\varepsilon =3.54\times 10^{-11}\;F\cdot m^{-1}$, as well as the mass density $\rho =1000\;\mathrm{kg}\cdot m^{-3}$.

It is clear that the increase in the ratio γ can increase the peak of the curve in Figure 6a. For a homogeneous film, which is γ=1, the specific energy generated per cycle reaches its peak of 2.70 J/g at $\Phi_{in}/H$ = 4.56 (V/μm). For a graded film with softer modulus (γ=0.5), compared to the homogeneous film, we have to input a larger voltage 6.22 (V/μm) in order to achieve the extreme value 1.60 J/g of the specific energy. It seems that a graded film with soft modulus cannot increase the ability of energy harvesting in a harvester. In contrast, a graded film with the stiffest modulus (γ=2) provides us with the largest specific energy 2.93 J/g at a relatively low input voltage 2.19 (V/μm). We apply the idea of low input voltage but large output energy by using a soft energy harvester with graded elastomers.

Similar to the amplification of specific energy, a graded film with stiff modulus (see γ=2 in Figure 6b) can also remarkably increase the output voltage of a soft energy harvester. We omit the detailed discussion of Figure 6b here. Based on the illustration in Figure 6, we find the answer that a graded film with stiff modulus can increase the two important values, the specific energy and the output voltage of a soft energy harvester.

### 5.2. Maximum Energy

At first, we distinguish *maximum energy* from specific energy. As shown in Figure 5, the specific energy in this paper is the area of the rectangle. In contrast, the maximum energy is the area enclosed by the five curves, such as the shaded area in each of the figures in Figure 4. The area of maximum energy, of course, includes the area of specific energy. Sometimes, the maximum energy is called the maximal specific energy.

In general, the specific energy is just the energy harvested by using a specific cycle. For example, the specific energy obtained in this paper is represented by a rectangle. Other shapes, such as the triangle, can be used to achieve different cycles by using different loading processes; therefore, different cycles can obtain different values of specific energy. However, the maximum energy of a soft energy harvester corresponds to only one particular value, that is the maximum value of the specific energy. This means that no matter what cycle you use to harvest the energy, the maximum energy is the limit of the specific energy.

**Figure 7:**
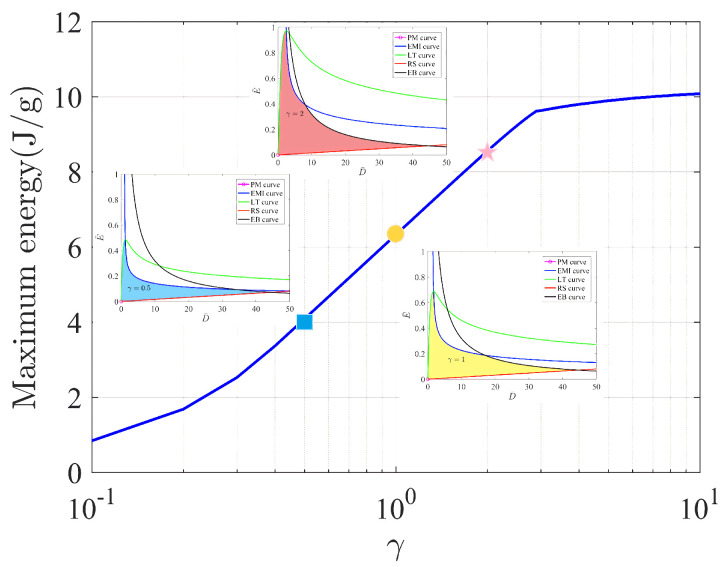
Maximum energy vs. the modulus ratio γ. The bule square ‘■’, the yellow circle ‘●’ and the pink star ‘★’ denote the maximum energy of a soft energy harvester with the ratio γ=0.5, γ=1 and γ=2, respectively.

In Figure 7, the maximum energy increases monotonically as the increase in modulus ratio occurs. For γ=1, which is the case of a homogeneous film, the maximum energy of a soft energy harvester is 6.3 J/g. If we increase the outer modulus of a graded film to γ=2, the maximum energy increases to 8.6 J/g. Moreover, the maximum energy can increase to more than 10.0 J/g when the modulus ratio γ is larger than 10. The graded film dramatically increases the ability of a soft energy harvester to achieve the maximum energy during the process of energy harvesting.

## 6. Conclusions

In this paper, we uses a graded elastomer to improve the functionality of a soft energy harvester subjected to electromechanical loads. We simply illustrate the working principle of a typical energy harvester made of soft dielectric films. To process the electromechanical behaviors in graded films, we revisit the equilibrium equations and four modes of failure in a circular film of homogeneous materials. We then formulated the governing equations in graded elastomers to show the effects of the graded modulus on the equilibrium states and the critical conditions for failure. Our results show that the graded materials can remarkably increase the specific energy, the output voltage and the maximum energy of a soft energy harvester. We hope the idea of using graded materials can be helpful in the design of powerful energy harvesters in the future.
